# Low Radiation X-rays: Benefiting People Globally by Reducing Cancer Risks

**DOI:** 10.7150/ijms.48050

**Published:** 2021-01-01

**Authors:** Fu-Jun Luan, Jun Zhang, Kin-Cheung Mak, Zhi-Heng Liu, Hai-Qiang Wang

**Affiliations:** 1 Department of Orthopaedics, Yongchuan Hospital of Chongqing Medical University, Chongqing City, P. R. China, 402160; 2 Department of Orthopaedics, Baoji Municipal Central Hospital, Baoji, Shaanxi, China, 721008; 3 Spine Central, Specialist Central, The Hong Kong Adventist Hospital, Hong Kong SAR, China; 4 Department of Orthopaedics, Chinese PLA No.986 Hospital, Fourth Military Medical University, Xi'an, Shaanxi Province, P. R. China, 710054; 5 Institute of Integrative Medicine, Shaanxi University of Chinese Medicine, Xi'an, Shaanxi Province, P. R. China, 712046

**Keywords:** radiation exposure, cancer, mortality, adolescent idiopathic scoliosis

## Abstract

Modern medical imaging facilitates the diagnosis and treatment of human diseases. However, few people are aware of the cons of radiation exposure from medical imaging. Emerging evidence reveals that cumulative doses of radiation exposure will increase the morbidity and mortality of pertaining cancer. As a special young population, patients with adolescent idiopathic scoliosis (AIS) suffer more radiation harms from repeated diagnostic imaging, most of which can be avoided in clinical practice. Accumulating evidence highlights reduced cancer risks of radiation exposure for AIS patients with low/zero radiation imaging modalities proposed, amongst which easy conversion from anterior-posterior (AP) to posterior-anterior (PA) projection for whole-spine radiographs should be stressed. It can greatly reduce radiation doses without compromising the quality of diagnostic imaging. Tight collimation combined with PA projection can further reduce radiation harms, and need to be spread to benefit people globally.

## Introduction

Nowadays, medical professionals are relying more and more on diagnostic imaging modalities, including traditional radiographs, ultrasound, CT, nuclear medicine, and MRI. Amongst these modalities, one of the common hallmarks exists as ionizing radiation, for radiographs, CT, and nuclear medicine.

Despite the fact that a single imaging for one patient might not be so harmful, the volume might be tremendous for cumulative radiation exposure over one patient with multiple times of imaging or cumulative imaging volume for global community. Over the past 3 decades, radiation exposure dose deriving from medical modalities (CT and nuclear medicine) equals or exceeds those from the natural radiation 1. In 2006, 380 million radiographic procedures and 18 million nuclear medicine procedures were performed in US. In comparison with 1980, the quantity represents a six-fold increase in annual per capita radiation exposure 2. Notably, high-dose radiation exposure elevates the lifetime cancer risks. Even low-dose radiation exposure from medical imaging might account up to 2% of cancer from coast to coast 3, in 680,000 Australians 4, in 178,604 patients' data in England, Wales and Scotland 5. Therefore, the issue of medical radiation not only affects the welfare of medical professionals and patients, but the entire environment and the welfare of people globally.

**Figure 1:**
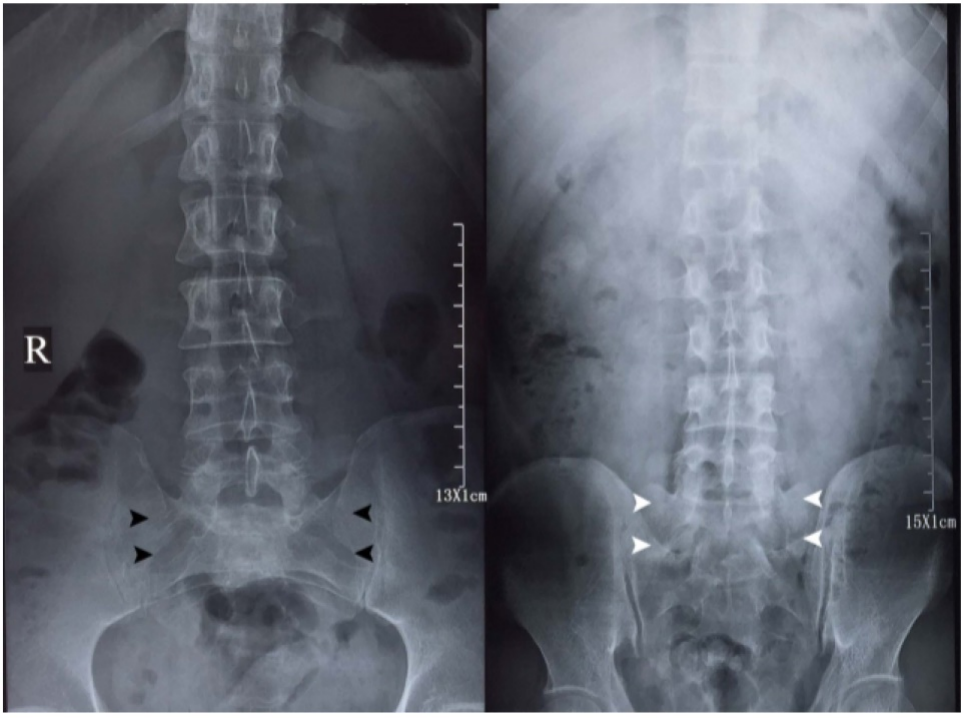
Anterior-posterior (AP, left) and posterior-anterior (PA, right) projection lumbar spine radiographs of a member of the studying group (Dr. Chi-Jiao Ma). The imaging quality of both radiographs is relatively comparable for diagnostic purpose of lumbar spine morphology. AP projection lumbar spine radiograph is characterized by sacral holes (black arrowheads). The hallmark of PA projection lumbar spine radiograph is the visualization of sacrum (white arrowheads).

**Figure 2:**
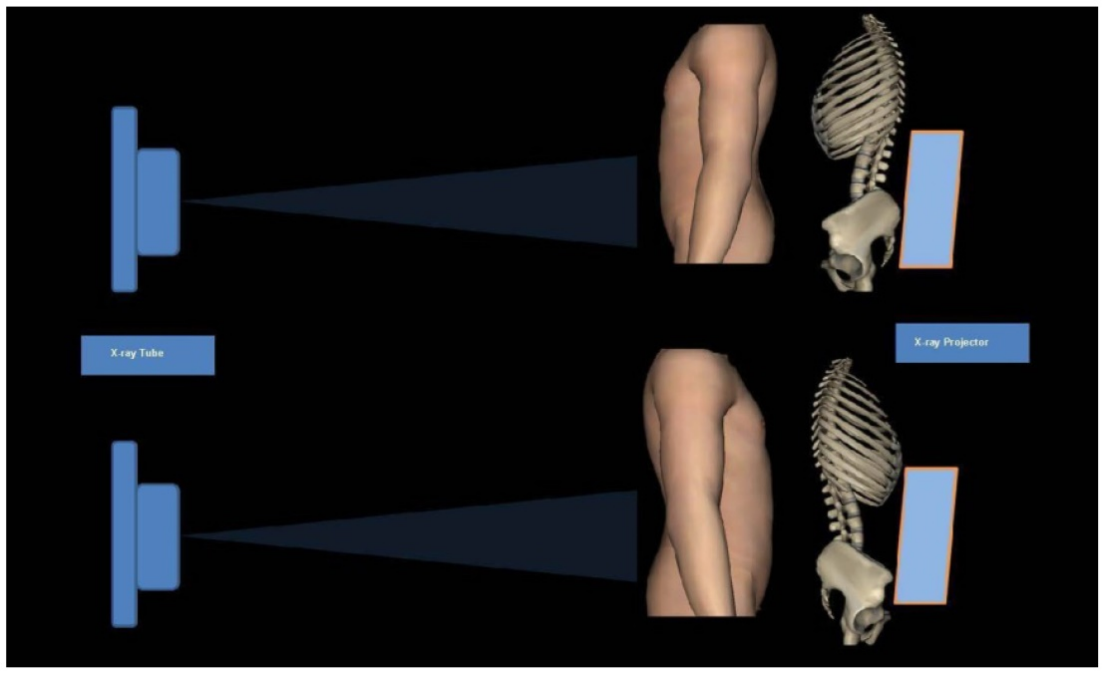
Schematic diagram of Anterior-posterior (AP, upper) and posterior-anterior (PA, lower) projection. Radiation sensitive tissues and organs localize in the front side of the body. Conventional AP projection will bring direct and high radiation dose on these organs and tissues. Notably, PA projection can reduce effective dose on these organs and tissues due to back shielding and absorption within human body.

Importantly and surprisingly, the level of radiation exposure knowledge is relatively low for medical diagnostic imaging amongst both the medical professionals, patients and the general public, based on accumulating lines of evidence, i.e., multi-nations (US and Finland) cross-section surveys 6-8. In clinical practice, low back pain is the most common disease second to influenza 9, both physicians and patients might concur on lumbar spine radiographs, without being aware of potential radiation exposure and the methodology for minimizing harmful effects, nor the red flag indicators 10. Anterior-posterior (AP) projection plus lateral views (Figure 1, Figure 2) of the lumbar spine will lead to a radiation dosage of 3.7 mSv 11, over three-fold to the recommended safe criterion as 1 mSv for general population annually 1. Therefore, there is an urgent need for medical professionals to propose the truth of radiation exposure and low radiation radiographic methodology, to benefit people globally.

## Search Strategy and Information Collection

Web of Science, PubMed, Scopus, CINAHL and EMBASE have been searched through May 31st 2020 using “scoliosis”, “adolescent idiopathic scoliosis”, or “AIS”. Citation tracking was conducted. Amongst searched abstracts and full-texts, pertaining information was collected and sub-divided into sections according to the major topics of the study.

## Lessons for Scientific Community: Adolescents with Scoliosis and Cancer Mortality

### Landscape of adolescent idiopathic scoliosis (AIS)

Amongst the entire adolescent population, there are a number of adolescents developing spinal curvature with unidentified causes. As such, AIS or late-onset idiopathic scoliosis is distinguishable from early-onset idiopathic scoliosis or children idiopathic scoliosis. The onset of AIS, which is prevalent in 2 to 4% of children, is between 10 to 18 years age 12. The threshold of diagnosing such spinal deformity is a spinal curvature over 10 degrees in the coronal plane, ideally in a whole-spine radiography from C7 to the iliac crest 13.

Currently, spinal surgeons commonly suggest a whole-spine AP projection radiograph and subsequently decisions of either watchful follow-up with frequent radiographs or aggressive intervention, including deformity correction surgery. However, tetrad issues might have been neglected, i.e., the etiology of AIS remaining elusive, the uncertainty of AIS screening as 3 steps according to US Preventive Services Task Force 14, the indefinable benefits of treatment on health and function, the optimistic natural history of AIS rather than pessimistic 15. Moreover, such kind of surgery comprises pedicle screw instrumentation, with the aid of instant fluoroscopic procedures with relatively high radiation doses, no matter C-arm 16, O-arm 17 or cone beam CT scans 18. Physicians and investigators come to realize the impact of management decisions on the radiation exposure and heath of the patients with AIS 19.

### Optimistic natural history of AIS

Persuasive evidence supports that the favorable natural history of patients with AIS is optimistic in terms of holistic view beyond the spine deformity *per se*. As for the most common disease, influenza is a typical disease of self-limiting in healthy individuals 20.

Weinstein and colleagues 15 summarized the natural history of untreated AIS patients over 50 years. One hundred and seventeen patients had a high level of productive ability without intervention. The milestone findings might bring lights and welfare for thousands and millions of adolescents. Sponseller 21 validated the outcome and further suggested only severe AIS patients (major thoracic curve: Cobb angle >80 degrees; major lumbar curve: Cobb angle >120 degrees) should be considered for surgical intervention. Given the low awareness of these insights, we distilled the essence of these articles and published through Chinese new media platform. Fortunately, more and more people realize that the natural prognosis of AIS is good. 22.

### Normal physical activity and pulmonary function of AIS

Scandinavia ScoliGeneS cohort (the Scoliosis and Genetics study in Scandinavia) comprised 1,278 idiopathic scoliosis adults diagnosed at various decades since 1960s (976 AIS cases; 360 untreated cases, 460 brace treated and 458 surgical treated cases). Comparing the physical activity of the cohort with normal people and within the cohort, Diarbakerli et al 23 suggested that patients with idiopathic scoliosis had similar physical activity level compared with normal people. AIS *per se* does not reduce physical activity ability.

In line with the optimistic natural history of AIS, Yaszay et al 24 noted that only severe thoracic curves with kyphosis affect pulmonary function.

### Painful price of neglected radiation exposure to AIS patients

Emerging evidence reflects the painful lessons and price for the medical community of repeated medical imaging and neglected great amount of cumulative radiation exposure.

It is well established that there are two types of side effects for radiation exposure upon the human body 1, including deterministic 25, 26 and stochastic effects, due to high and low (cumulative) dose exposure, respectively. Fukushima nuclear crisis in 2011 and radiation skin injuries are typical radiation effect events; whereas increased cancer risks of children/adolescents with pediatric radiation exposure are typical lessons for medical diagnostic imaging. In contrast with the productive and functional lives of untreated AIS patients, treated AIS patients experienced numerous AP projected X-rays, unexpected high cumulative radiation doses, and reported increased cancer and death risks. Several lines of evidence supported the lessons for medical community, the harms upon thousands and millions of adolescents, i.e., Denmark AIS cohort with 25 years follow-up 27, 28, US AIS cohort with 47 years follow-up 29, 30, and Canada AIS cohort with 11-30 years follow-up^31^-^33^. These cohort studies focused on not only cancer and mortality risks among AIS patients; but also the reproductive issue.

According to Simony and colleagues 27, the ionizing radiation dose for a whole-spine radiograph was 0.8-1.4 mSv per time and 2.4-5.6 mSv per year (Table 1). Analyzing radiographic data of 205 patients from the cohort with a mean period of 24.5 years (range: 22-31 years); Simony and colleagues demonstrated 16 whole-spine radiographs were taken on average for AIS treatment. The cancer rate of these patients was 5 times higher than age-matched general Danish population. Collectively, cumulative radiation exposure is harmful for treated AIS patients, especially in terms of breast cancer.

US scoliosis cohort 30 comprised 5,573 women with mainly scoliosis, diagnosed between 1912 and 1965. It should be stressed that the awareness of radiation risks among medical community evolved with the practice of radiology and improvement for reducing radiation means. During the early decades of twentieth century, radiation risks were not well characterized. As a consequence, both patients and medical personnel were exposed to harmful radiation, even fatal dose 34.

By the end of 2004, Ronckers and colleagues 30 collected and analyzed radiation, cancer and mortality data via medical records and public health resources. In total, 5,573 women with scoliosis received X-ray radiation for 137,711 procedures before 20s. The mean number of radiographs per patient with exposure to the breast was 22.9 (range: 0-533).The estimated average cumulative dose of breast was 109 mGy.

Forty-seven years later, the average age of the cohort was 58 years old and 1,527 women died. Amongst the 355 patients died from cancer, breast cancer contributed mostly (112 cases). The overall mortality risk was 46% higher than the normal population. Furthermore, risk of breast cancer was linked with the number of X-rays upon breast.

One new study 35 noted the relation between risks of cancer and mortality of patients with scoliosis and radiation exposure. Based on 35,641 participants from 1912 to 1990 with over 20 years' follow-up, repeated full-spine radiographs caused elevated risks of cancer, breast cancer and cancer mortality for scoliosis patients. Low or free radiation means should be recommended to investigate the evolution of AIS.

Canada AIS cohort 31 (named as Ste-Justine AIS Cohort 36) included 2,092 AIS patients referred from 1960 to 1979 with AP projection X-ray. In 1990, Goldberg and colleagues 31 analyzed the cohort with 1,292 females involved in terms of reproductive issues. In average, the dose to the ovaries was 9.25 mGy. In comparison with 1,134 females from the same area, risks of unsatisfactory reproductive events in AIS cohort were higher, in terms of unsuccessful attempts at pregnancy, spontaneous abortions, low birth-weight, congenital malformation, and stillbirths. Furthermore, another AIS cohort study 37 noted that patients with scoliosis had a higher rate of premature births than expected with 846 women diagnosed during 1927-1965.

**Figure 3:**
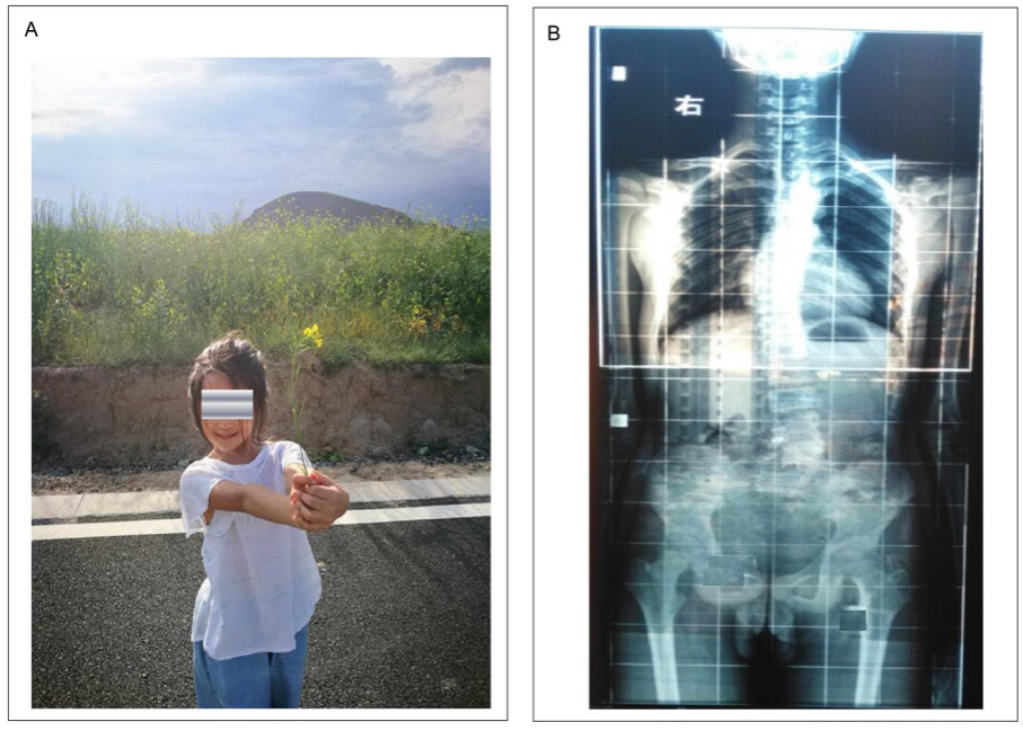
Left: A young female from China with AIS. She was diagnosed AIS aged 20 with a Cobb angle of 30 degrees. Unawareness of the truth on AIS and being afraid of negative effects on her daily life, her parents had to undergo surgery as suggested by a spinal surgeon. Right: Typical whole-spine radiograph of a male with spinal scoliosis. Note AP projection with loose collimation and wide radiation field. The patient and his parents were not aware of radiation exposure.

Besides, surgical treated AIS patients have to face a series of issues, including excessive bleeding and blood transfusion 38, pancreatic fractures 39, reservation of metal implants within the body lifelong with elevated levels of metal irons in serum and hair 40, and allergies or hypersensitivities reactions to implants 41, 42 (Figure 3).

Collectively, profound analyses of 8,716 AIS patients from various cohorts and time periods consistently demonstrated the neglected cumulative radiation dose, adverse pregnancy outcomes, and increased cancer and mortality risks during diagnosis and treatment for AIS using traditional AP projection radiographs.

## Low Radiation Medical Imaging: Transfer from AP to PA (Posterior-Anterior)

### Human tissue orientation hallmarks

In general, human tissues/organs is characterized by their orientation. For normal persons, the heart localizes in the left, whereas the liver is in the contrary side in terms of orientation. In contrast, most radiosensitive organs localize in the front body, including eyes, thyroid gland, breast, and gonad. Notably, the spine and pelvic are located in the back of the body. Rarely, persons with heart in the right side are referred as dextrocardia, visceral mirror inversion or situs Inversus 43. Radiation sensitivity of organs is reflected as weighting factors (in total: 1 for the body), the highest organs include breast (0.12), stomach and colon (0.12), gonad (0.08), bladder (0.12), liver and thyroid gland (0.04) 1.

As such, photons within X-rays enter the body for AP projection radiographs will result in a mass of radiation absorbed by the aforementioned radiosensitive organs in the front, than that for PA projection radiographs. In particular, repeated radiographs would lead to potential harms upon these organs, even cancer risks.

### PA projection transition for the spine

For medical imaging, it is well known that ALARA principle (as low as reasonably achievable) should be conformed to. Correspondingly, medical community has the Canadian C-spine rule for radiography in suspected cervical spine injured patients 44.

In early 1980s, Gray et al 45 revealed that PA projection can reduce radiation harms to radiation-sensitive organs while maintaining the quality of spine imaging. Thereafter, the significance of the simple transfer and benefits upon patients has been recognized by limited number of medical professionals. Monte Carlo effective dose simulation indicates that AP projection at the thoracic spine can result in an excess dose to breast: 543.3% for 10-year-old Children, 597.0% for adults. Notably, the effective dose of PA projection can decrease over 64% for 10-year-old children and 65% for adults.

For lateral radiographs of the spine, there are two projection options, i.e., right-to-left and left-to-right projection. Based on the orientation of human radiation-sensitive organs, right-to-left lateral projection angles can reduce effective dose 46 (reported in 2016). The projection is suitable for the cervical, thoracic and lumbar spine.

Another important but neglected issue regarding radiographic techniques is X-ray beam collimation over diagnostic interest area, referring to the part of a patient that physicians care. In clinical practice, loose collimation of a radiograph will create over three times higher radiation doses to sensitive organs than tight collimation 47.

It should be stressed that these established low-dose methodological lines of evidence have not been applied in clinical practice widely, based on available applied reports (the Netherland 48, and Greece 42), as well as un-applied reports (French spinal surgeons 39, and Korean neurosurgeons 49). Notwithstanding radiation exposure and pertaining cancer risks for patients with AIS were reported in the updated US Preventive Services Task Force Recommendation 14, PA projection with tight collimation technique has not been well documented.

### PA projection transition for the pelvic, abdomen and clavicle

The easy conversion from AP to PA projection with great significance could be used for other parts of the body, based on the same principle of the orientation hallmarks of organs of the human body.

As for the abdomen, Marshall and colleagues 50 made a comparison of radiation dose of the abdomen using AP and PA projection in terms of effective dose to individual radiosensitive organs. Subsequently, investigators from Ireland 51 further consolidated PA projection radiograph as significant dose-reducing methodology. Due to differences in simulation and calculation details, dose reduction rates varied with consistently supporting the advantages of PA projection. As for the pelvis, Brennan 52 provided evidence supporting PA projection with reduced radiation dose. Subsequent studies paid attention to the transition greatly 53.

As for the clavicle, Sharr and Mohammed^54^ identified PA 15 degrees caudal view (PA15 caudal projection) of the clavicle as more accurate in comparison with the standard AP 15 degrees cephalad view in terms of length and clavicular alignment. The true length of skeletal clavicle is 124 mm. Under AP15 view, the length of the clavicle was enlarged to 149 mm with magnification of 15%. Under PA15 view, the length of the clavicle was 130 mm with magnification of 5%. Therefore, PA 15 degrees caudal view of the clavicle was recommended more accurately. Furthermore, Mc Entee and Kinsella 55 compared image quality and dose during PA and AP view of the clavicle. Whereas PA and PA15 caudal projections significantly reduced in the dose to the eye, breast and thyroid, reductions in image quality were non-significant.

### Other imaging methods for scoliosis with low/zero radiation

The micro-dose EOS (EOS imaging, Paris, France) can be used to evaluate scoliosis in adolescents/children with high a quality image. Radiation exposure of micro-dose method was a 45-fold reduction compared with conventional radiographs 56. Rasterstereography detects the 3D spine deformity using the topography of the surface of the patient's back. Tabard-Fougere and colleagues 57 found the rasterstereography can assess AIS patients with a good validity compared with radiography. Scolioscan, which uses a 3D ultrasound volume projection imaging method, can be used to assess scoliosis. Zheng and colleagues 58 concluded that scolioscan was dependable to measure coronal deformity for AIS patients with radiation-free and low-cost.

## Summary

Notwithstanding modern medical imaging facilitates the diagnosis and treatment of human diseases, significant side effects emerge with negligence. Amongst these effects, radiation exposure quietly harms the health and welfare of people globally. The increase in natural radiation of the earth has been largely ascribed from medical imaging, including X-ray, CT, interventional diagnosis and treatment, and radiotherapy. Whereas medical community is partly aware of the harms of high-dose radiation, the cumulative radiation exposure deriving from repeated radiographs and fluoroscopy have not been well understood by medical professionals, patients and the public. One of the typical lessons for medical community is cumulative large amount of radiation dose among AIS patients. Profound analyses of 9,994 AIS patients from various cohorts consistently demonstrated the neglected cumulative radiation dose, adverse pregnancy outcomes, increased cancer and mortality risks during diagnosis and treatment for AIS with AP projection radiographs. Importantly, the natural history and physical activity level of AIS are optimistic. A simple transition from AP to PA projection radiographs has been validated as effective in reducing radiation exposure to the eye, thyroid, breast and gonad. PA projection radiographs with tight collimation could be applied for whole-spine, cervical, thoracic and lumbar spine, pelvis, abdomen and the clavicle. Collectively, we propose the low-radiation PA radiographic methodology should be spread widely, consequently benefiting people globally.

## Figures and Tables

**Table 1:** AIS Cohorts with long-term studying

Regions and Investigators	Cohorts	Analyzing time span	Radiographic and radiation hallmarks	Radiation dose (mSv)	Main findings
**A Untreated AIS with a good prognosis**					
Weinstein et al from US Ref 15	Iowa prospective AIS Cohort 444 AIS cases between 1932-1948 117 untreated cases survived in 1992 62 age and gender-matched volunteers	Average: 51 years Range: 44-61 years	PA projection at later follow-up at standing position Collimation unavailable Details of radiographic history and radiation exposure unavailable	-	Untreated adults with AIS are productive and functional at a high level.
Diarbakerli et al from Sweden Ref 23	Scandinavia ScoliGeneS cohort 1,278 adults with idiopathic scoliosis diagnosed at various decades since 1960s 976 AIS cases	Median years from surgery: 18.9 years Median years from brace cession: 27.4 years	Details of radiographic history and radiation exposure unavailable	-	AIS does not affect physical activity level.
**B Over-treated AIS with a poor prognosis**					
Simony et al from Denmark Ref 39, 40	Denmark retrospective AIS Cohort 215 AIS cases treated with brace or surgery between 1983-1990 205 analyzed cases	Average: 24.5 years Range: 22-31 years	AP projection Loose collimation 16 radiographs/case Radiation exposure: 2.4-5.6mSv/year	12.8-22.4/case 2.4-5.6/year	The overall cancer rate in these patients was 5 times higher than age-matched Danish population.
Ronckers et al from US Ref 27, 28	US Scoliosis Cohort from 14 medical centers 5573 female patients diagnosed during 1912-1965 5513 analyzed cases	Median: 47 years Range: 0-91.5 years	Projection: AP (64.3%) PA (1.3%) 137000 diagnostic procedures in total Radiation exposure to breast: 22.9 radiographs/case	Mean cumulative dose: 100-150mGy breast dose: 109mGy (Max: 1700mGy) Lung: 41mGy Bone marrow: 10mGy Thyroid: 74mGy	Mortality from breast cancer significantly increased.
Goldberg et al from Canada Ref 30	Ste-Justine AIS Cohort 2,092 cases (1793 females) referred from 1960 to 1979 1,292 analyzed women with 1134 normal women as control	Studying in 1990	Most AP projection Loose collimation	Mean dose to the ovaries: 9.25mGy	Risks of unsatisfactory reproductive events in AIS cohort were higher than normal women.
Visscher et al from US Ref 31	1,409 persons diagnosed with scoliosis between 1927 and 1965 in 846 women analyzed, 615 AIS women	Studying in 1985	AP projection Loose collimation	-	Scoliosis patients had more premature births than expected.
Hoffman et al from US Ref 32	1,645 persons diagnosed with scoliosis between 1935-1965 in Minneapolis and St. Paul, Minnesota 1,030 women analyzed	Studying in 1983-1986 Average: 26 years	AP projection Loose collimation Over 40,000 x-rays, average: 41.5 x-rays/case	Average dose to breast 128mGy	Frequent exposure to low-level diagnostic radiation increases the risk of breast cancer.
**C PA projection with predominance**					
Levy et al from Canada Ref 33	Ste-Justine AIS Cohort 2,092 AIS cases (1751 females) referred between 1965 and 1979 2,039 cases analyzed	Studying in 1982	Most AP projection (76%); PA (3%) Loose collimation Average X-rays: 12/female; 10/male	Mean cumulative dose: AP: 29mGy PA: 5.5mGy	AP replaced by PA view could result in 3 to 7 fold reduction in cumulative dose to the thyroid gland and female breast, reducing breast and thyroid cancer risk.

AIS: Adolescent idiopathic scoliosis. AIS Cohort: 13063 cases.
